# Psycho-Historical Contextualization for Music and Visual Works: A Literature Review and Comparison Between Artistic Mediums

**DOI:** 10.3389/fpsyg.2019.00182

**Published:** 2019-02-19

**Authors:** Anthony Chmiel, Emery Schubert

**Affiliations:** Empirical Musicology Laboratory, School of the Arts and Media, University of New South Wales, Sydney, NSW, Australia

**Keywords:** appreciation, preference, music, visual art, context, program notes

## Abstract

A significant contribution to the literature on aesthetics in the last decade has been Bullot and Reber's ecologically-driven *psycho-historical framework for the science of art appreciation* (PHF). The framework proposes that the presence of contextualizing information accompanying an artwork will impart a substantial impact on appreciation for it, which is accessible through understanding of the *causal information* surrounding the work. Artistic understanding is outlined in terms of three hierarchical “modes” of appreciation. This paper tested a simplified hypothesis drawn from the PHF, using results reported in the existing literature. As Bullot and Reber note that such a framework is relevant for any artistic medium containing causal information, results were drawn from literature concerned with either music or visual works. Our review identified 34 studies that reported results of appreciation (or equivalent) as a dependent variable, while manipulating contextual/historical information for the stimuli as an independent variable. Overall the results were consistent across the two artistic mediums: 9 experiments (26%) produced strong support for the PHF, 6 experiments (18%) produced inconclusive results, and 19 experiments (56%) produced no support for the PHF. We concluded that the majority of the reviewed literature does not support the simplified PHF hypothesis for either medium. However, we also discuss a number of limitations surrounding these studies which may have produced a substantial impact on the categorization results: small sample sizes in some studies, difficulty in translating philosophically-based theory into empirical practice, and interactions with variables such as exposure and “unusualness.”

## Introduction

Empirical investigations into the effects of accompanying information for both music and visual art works have become increasingly prevalent, and this line of study should come as little surprise considering the frequency of contextualizing notes at performances and exhibits. However, there remains little consensus as to exactly how much of an impact accompanying information has on our appreciation for these mediums. This paper investigates a framework formulated for various artforms, Bullot and Reber's (2013a) *psycho-historical framework for the science of art appreciation* (henceforth PHF), and examines the hypothesis drawn from the framework. We perform a comprehensive review of extant, relevant results reported for music and visual works in the context of the PHF, and accordingly we hope to gain more insight into the overall predictive utility of the PHF for each of the two mediums (music and visual art).

## Outline of the Psycho-historical Framework for the Science of Art Appreciation

In the PHF Bullot and Reber suggest two opposing, yet not incompatible, methods of approaching aesthetics for artistic works: a *psychological approach*, and a *historical approach*. Whereas the psychological approach is centered on mental and neural explanations, the historical approach focuses on a respondent's *art-historical sensitivity* to a work, referring to their ability to produce a historically-informed response. In other words, this sensitivity encapsulates the appreciator's ability to process the historical events and artist actions surrounding a work. The PHF invokes a philosophical standpoint known as a*esthetic contextualism* that prioritizes the impact of such contextual knowledge in an appreciator's identification, appreciation, understanding, and evaluation of a work. Bullot and Reber (2013a, p. 125) note that as most “contextualists” reject aesthetic approaches that do not account for the role of *causal historical information* embedded within artworks, this contextualist objection extends to a rejection of most psychological and neuroaesthetic explanations of appreciation. Causal historical information refers to historical data carried within the features of a work, such as the outcome of deliberate or unintentional actions, and can be impacted by people, cultural influences, political events, market-place factors, and the like. As a number of artistic mediums such as music, literature, dance, and visual and cinematic works carry causal information, the framework is transferable to a number of artistic areas (Bullot and Reber, 2013a, p. 127).

The PHF consists of three hierarchical “modes” of appreciation, with subsequent modes enabling a deeper historical understanding of a work and thereby impacting appreciation for it (p. 135). The first mode, *basic exposure*, requires no knowledge of the art-historical context of a stimulus as appreciation is based on observable features, although repeated exposures to a stimulus while in this mode may enable an individual to develop their historical sensitivity regardless of whether or not they receive information from an external source. This mode is regarded as elementary in comparison to the later modes, and is likened to the majority of exposures used in psychological experiments. Once a respondent begins to reason about a work, such as the functions of the work through its perceivable qualities, origins, and the intentions of those who produced it, they may adopt the *artistic design stance*, the second hierarchical mode. This stance is described as “far from historically shallow” (Bullot and Reber, 2013a, p. 129), and encompasses development of sensitivity through reasoning in terms of authorship, style, craftmanship and reception. The final mode, *artistic understanding*, is linked to the highest amount of art-historical sensitivity and proficiency, and allows theory-based reasoning and evaluations of status, function, merit, and value in comparison to other works. Importantly, while the PHF hypothesizes that understanding will produce an impact on appreciation, this impact is not necessarily hypothesized as being positive in nature (see *aesthetic-artistic confound*, Bullot and Reber, 2013b, 2017). As an example, consider a person that is exposed to music by Richard Wagner, but is unaware of any details of the composer's life. If this listener was subsequently informed of Wagner's well-documented anti-Semitic stance, it is conceivable that this information might produce a negative impact on their appreciation for the music (depending on the listener's own sensitivity to the subject). As such, any evaluation of the PHF must take this facet of the framework into account.

## Aims of the Current Study

We wanted to examine whether studies reporting appreciation for visual and music stimuli have observed results that support the hypothesis of the PHF. For the purposes of investigating extant empirical research, we use a simplified hypothesis drawn from the PHF (henceforth PHF hypothesis), that posits that contextual information provided about a work of art will increase understanding of an artwork, and produce a significant impact (either positive or negative) on appreciation for it. Therefore, our approach was to perform a comprehensive literature review on each of the mediums. To our knowledge no previous study has explicitly examined the PHF in terms of music stimuli, and no comprehensive analysis of reported data across multiple studies exists.

## Methods and Materials

### Design

As the PHF hypothesis is based on understanding of contextual elements surrounding a work, one approach would be to limit such a review to studies reporting ratings of both appreciation *and* understanding (that is, two distinct variables). Such an approach would allow direct examination of the relationship between the two variables, and could enable detailed investigation into the three modes of appreciation outlined by Bullot and Reber by manipulating them as independent variables or variable levels. However, this would also severely limit the number of studies that could be included. In response to this we took a more liberal approach by assuming that any exposure to additional contextual information enables progression to either of the two later modes of the PHF, although it must be noted that this is a necessarily simplified interpretation of “historical sensitivity.” Which of the two later modes might be reached is not explicitly examined; this investigation could therefore be viewed as comparing the *basic exposure* mode with the later two modes of the PHF, which are collapsed. We also perform a separate examination on the subset of studies that report ratings of both appreciation *and* understanding (see section Main Findings).

In this work we refer to appreciation as an umbrella term for aesthetic responses to artistic works, encapsulating preference, liking, enjoyment, pleasingness, appealingness, and the like. A number of variables not included in this definition—such as beauty, color, interest, and meaningfulness—could be seen to hold a strong relationship with the appreciation of artistic works. However, in the interest of producing a focused empirical review we decided to limit the range of dependent variables, although subsequent investigations may benefit from incorporating these additional variables. Further more, we decided not to include studies that only manipulated perceived effort, craftmanship, or quality as independent variables in this review because they relied on manipulation of information rather than type and amount of information. Therefore a number of studies were excluded (e.g., Duerksen, 1972; Kruger et al., 2004; Kirk et al., 2009; Steinbeis and Koelsch, 2009; Jucker et al., 2014; Kroger and Margulis, 2016; Anglada-Tort and Müllensiefen, 2017). Bullot and Reber draw a distinction between the inclusion criteria of the PHF and such experimental approaches as well (2013a, p. 133). Addtionally, Millis (2001, experiment 2) investigated the influence of titles upon aesthetic experience of images, but was excluded from this review; while participants were given accompanying information (the titles), it was unrelated to the stimuli *and* the participants were informed of this irrelevance. Millis asked the participants to ignore the information, and instead aimed to examine the effects of quasi-subliminal information.

### Procedure

Literature for each of the two mediums (visual and music) were examined separately. Literature was identified using various combinations of general and keyword searches, such as the dependent variables listed in section Design, and “visual artworks,” “music,” “contextual information,” “program notes,” and the like. Searches were performed in *Google Scholar, Répertoire International de Littérature Musicale* (RILM), and *PsycINFO*. Analysis of articles cited in these papers were also accessed to encompass a broad review of possible papers that satisfied the inclusion criteria. The inclusion criteria for each medium were that: (1) at least one dependent variable was a kind of appreciation (such as enjoyment, liking, preference, and so on; see section Design); (2) the amount of contextualizing information (independent variable) was manipulated. For example, one condition might receive no contextual information, or substantially less contextual information such as only a title, in comparison to a second condition receiving a detailed description of circumstances surrounding the creation of the work. In such a circumstance we assume that respondents who are exposed to additional contextual information progress to one of the two later stages of the PHF.

Our review categorized studies into one of the following:

- Studies in which the results produce strong support for the PHF hypothesis. An increased amount of information accompanying a stimulus must produce a statistically significant difference for ratings of appreciation in comparison to less information. We set the criterion for “statistically significant” to comparisons that produced results which reject the null hypothesis with a Type I error of less than 5% (i.e., *p* < 0.05, with corrected value if required), applying the same criterion across all studies, based on the relevant statistics reported by their authors or ascertained. As the PHF hypothesis posits that impact on appreciation can be positive or negative (see section Outline of the Psycho-Historical Framework for the Science of Art Appreciation), we denote negative significant differences with A^−^;
- Studies that have inconclusive results with regard to the PHF hypothesis. For example, studies consisting of mixed results within the same experiment, with some results supporting the PHF hypothesis while other results in the same experiment do not. Studies categorized as “B” were subjected to additional analysis;
- Studies in which the results do not support the PHF hypothesis (i.e., those reporting non-significant results).

A small number of studies collected ratings explicitly for understanding in addition to ratings concerned with appreciation. Given the importance of the variable “understanding” in explicitly defining the later two modes of the PHF (see section Design), this variable was also assessed by us in terms of the strength of the relationship found in connection with the conditions used. For example, whether or not significant increases in understanding were reported for a contextualized condition, vs. a condition receiving no contextualization. Therefore, understanding was separately categorized in a similar manner to appreciation (although denoted with lowercase categories):

- Studies in which the contextualized conditions produce statistically significant higher ratings of understanding compared to conditions receiving less or no contextualization;
- Studies with inconclusive results in regards to contextualization and understanding. Studies categorized as “b” were subjected to additional analysis;
- Studies in which none of the highly-contextualized conditions produce significantly higher ratings of understanding compared to conditions receiving less or no contextualization.

## Results

**Figure 1:**
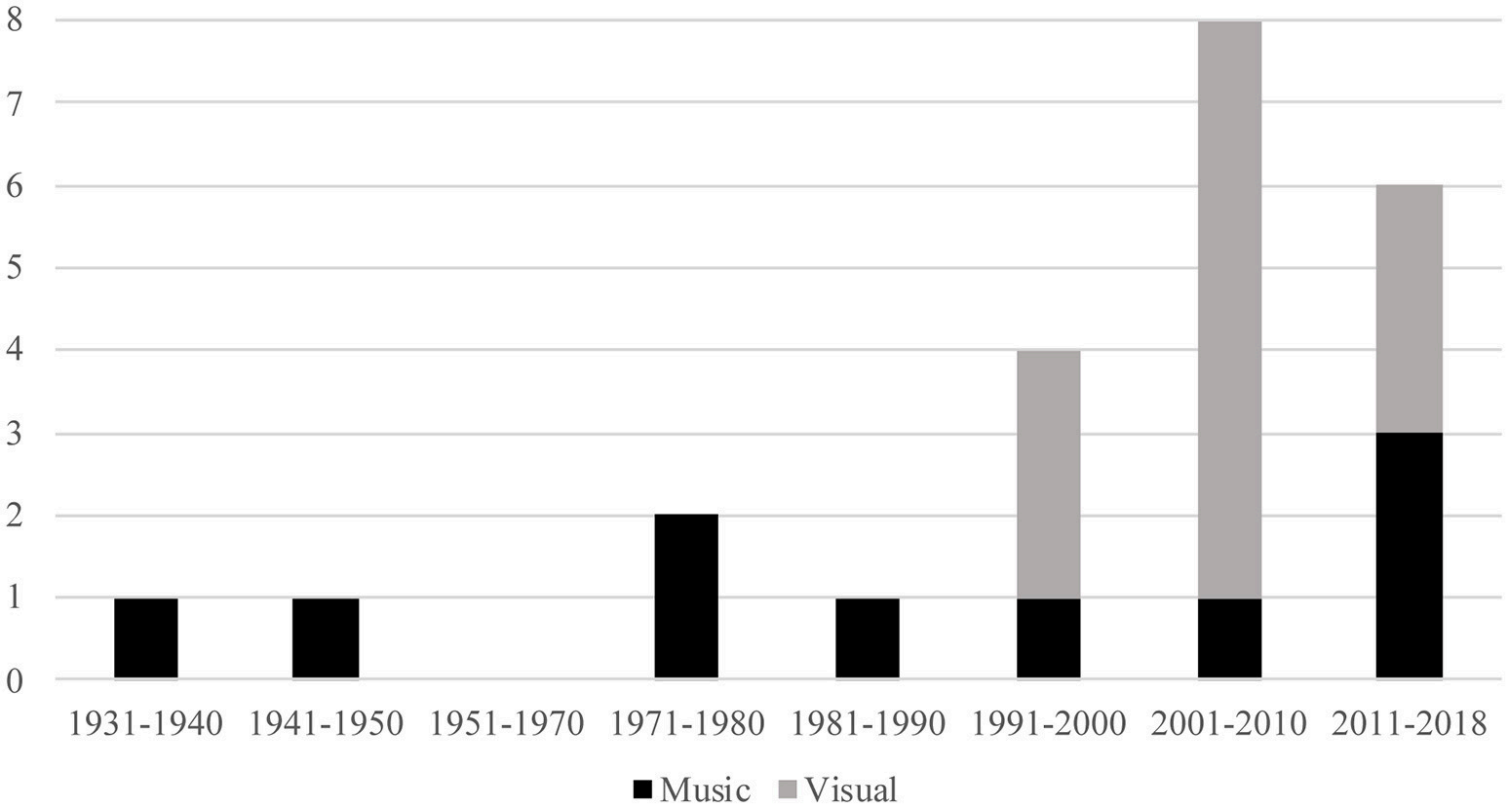
Count of studies included in the review, grouped by stimulus type and time period.

Thirty-four experiments met the inclusion criteria (from 23 separate publications). Twenty-two experiments investigated visual stimuli and 12 investigated music stimuli. Before categorization, we re-analyzed the data used in two publications (Damon, 1933; Swami, 2013). The reasons for these re-analyses, and the re-analyzed data can be found in the Supplementary Material. Experiment details and categorizations for reviewed studies are listed in Table 1. The 23 publications ranged in published year from 1933 to 2018. Only 2 studies were published prior to 1970, both of which contained music stimuli, and including the two studies published prior to 1970, 5 studies were published prior to 1990. As before, all of these contained music stimuli (that is, all of the visual studies included in this review were published after 1989). Overall, 9 studies were published prior to 2000 (6 of which contained music studies). Therefore, the majority of papers in this review (14 studies; 61%) were reported in the last two decades, suggesting that the topic has become increasingly popular. The distribution of studies by publication year and stimulus type can be observed in Figure 1.

**Table 1:** Tabulation of literature reviewed on the influence of contextual information upon appreciation of artworks, with results of reported primary dependent variables categorized as per section procedure.

**Author(s) and year**	**Stimuli**	**Label for dependent variable of interest**	**Result categorization^a^^,^^b^**	**Understanding categorization^a^^,^^c^**	**Levels of independent variable(s)^a^**	**N^a^^,^^d^**	**Exposures^a^**	**Comments^e^^,^^f^**
**VISUAL STIMULI**								
Belke et al., 2006	36 abstract paintings	Liking	C		None, stylistic	60	2	
Bordens, 2010	Color pictures of 8 paintings and 8 sculptures from 4 styles (2 representational, 2 abstract)	Liking	C		General introductory, specific historical	172	1	Art styles were dada, impressionist, outsider, and renaissance art
Cleeremans et al., 2016	12 abstract paintings	Preference	C		None, name of the artist	40	1	As *p* > 0.05 for “Presence of name” this study was categorized as “C”
Cupchik et al., 1994	3 abstract figurative artwork installations; 12 slides of abstract or rhetorical artwork	Pleasingness	C; C		Title, elaborated title with mood, stylistic; Mere description, stylistic, broader social context	48; 48	2; 2	In Exp. 1, each installation comprised of a painting with a sculpture set in front of it
Jucker et al., 2014	12 semi-abstract to abstract artworks (10 paintings, 1 drawing, 1 collage)	Liking	C; C^i^	a (for first categorization—Exp. 2a— only)	None, descriptive title, elaborative title, relevant title (suggesting precise interpretation)	212; 114	1	Exp. 2a and 2b only. ^i^Exp. 2b is categorized as “C.” This was because ratings for the “relevant” condition were only significantly higher than the “descriptive” condition; “relevant” was not rated higher than “control” or “elaborative.” However, this could also be interpreted as “B”
Leder et al., 2006	48 images of paintings (24 abstract, 24 representational); 24 abstract paintings	Liking	C; C	b; b	None, descriptive title, elaborative title; Descriptive title, elaborative title	48; 48	1; 2	In Exp. 2 the first exposure was only for 1 s, and the second exposure only for 10 s
Millis, 2001	30 representational artworks (15 colored illustrations, 15 black and white photographs); 30 colored illustrations (15 abstract, 15 representational)	Aesthetic experience^ii^	A; C	a; a	None, descriptive title, elaborative title	166; 92	2; 1; 1	Exp. 1 includes the replication experiment. Exp. 2 is not included (see main text). ^ii^Participant mean for enjoyment, interest, evoked emotions, and elicited thoughts were collapsed into the variable “aesthetic experience”
Russell and Milne, 1997	20 semi-abstract to abstract paintings	Pleasingness	C		None, title	160	1	Exp. 1 only
Russell, 2003	12 semi-abstract to abstract paintings	Pleasingness	C; A		None, title and artist, descriptive; None, descriptive	120; 45	1; 2	Exp. 1 used a between-subjects design; Exp. 2 used a within-subjects design
Smith et al., 2006	4 paintings (2 impressionist, 2 modern)	Evaluative subscale^iii^	C		None, elaborative	152	1	^iii^Evaluative subscale consisted of ratings of pleasingness, interest, appeal, inspiration, and invitingness
Specht, 2010	Images of abstract and representational works; Abstract and representational paintings	Liking	B; B		None, artist's statement on their own work; None, artist's statement switched from the opposite work^g^	72; 26	2; 2	The methodology used in both Exps. does not account for any effects of mere exposure upon liking. Exp. 1: only representational stimuli produced significantly higher ratings with the artist's statement. Exp. 2: only abstract stimuli produced significantly higher ratings with the (incorrect)^g^ artist's statement
Swami, 2013	12 surrealist paintings (Exp. 1 and 3); 8 abstract and 8 representational paintings	Aesthetic appreciation (mean of liking and interest)	A; B; A	a; b; a	None, title, broadly descriptive, content-specific; None, contextual; None, content-specific; 3 content-irrelevant conditions	155; 140; 257	1	For Exp. 2, only abstract stimuli produced significantly higher ratings when accompanied by contextual information. See Supplementary Material for further details
Temme, 1992	40 color slides of Seventeenth and Nineteenth Century Dutch paintings—half ambiguous, half representational; 12 paintings from the Seventeenth century	Agreeability; Aesthetic appreciation	B; A^—^		None, description before rating, description after rating; Exp. 4 used 4 levels of increasing amounts of information	172; 160	1	Exp. 1 and 4 only. Exp. 1 significant for ambiguous stimuli only. Exp. 3 was excluded as participants only rated preference for accompanying information labels—they did not rate preference for the artworks
**MUSIC STIMULI**								
Anglada-Tort et al., 2018	30 s excerpts of dance and electronic music	Liking	A		None, title^iv^	93^v^	1	Exp. 2 only. ^iv^Titles were valenced, but this review collapses all valences. ^v^Ratings were compared to those from Herzog et al. (2017)
Bradley, 1972	24 contemporary art compositions representing tonal, polytonal, atonal, and electronic music	Preference	A^vi^		None, title and composer only, special training and experience in listening analytically	820+	2, 5, 16^vi^	*N* is not reported for the control condition. ^vi^As the experimental conditions received additional exposures, any increase in preference could be attributed to this. See main text for details
Damon, 1933	12 pieces, evenly chosen from orchestral, solo violin, and vocal ensemble music	Enjoyment	B		None, title, elaborative	120	2	Exp. 1 only. The results reported here summarize the inferential tests detailed in the Supplementary Material
Halpern, 1992	3 classical pieces (Bach, Debussy Poulenc) and a popular piece (Jimmy Cliff)	Enjoyment	B	b	None, descriptive, historical	45	1	Only the Debussy piece produced a significant result, for which those in the historical condition rated enjoyment highest
Margulis, 2010	24 excerpts (45 s) of Beethoven string quartets	Enjoyment	A^—^; C		None, dramatic description, structural description	16; 11	1; 2	Both Exps. used a within-subjects design. For Exp. 2, information was provided at the First exposure, without enjoyment ratings. Enjoyment was only rated after the second exposure
Margulis et al., 2015	An hour-long performance of “quintessentially American” immigrant music such as bluegrass	Enjoyment plus two related questions	C		Placebo note only containing information on the performance venue, contextual	506	1	The two related questions asked whether participants would like to attend a similar performance, and whether or not it was one of the best shows they had seen
Prince, 1974	40 excerpts, with 10 from each of baroque, classical, romantic, and Twentieth Century music	Liking	C		No listening classes, guided analytical listening classes for baroque, guided analytical listening classes for Twentieth Century music	342	2	Used a pre-test vs. post-test design, with a training procedure of 12 weeks in between
Rigg, 1948	6 pieces of operatic and orchestral music (Wagner, Beethoven, Sibelius, Franck)	Liking	A		None, “negative propaganda,” “positive propaganda”	164	2	The “negative propaganda” associated the music with Nazi members and ideals. Inferential tests were not performed on the data, and *SD* values are not reported
Vuoskoski and Eerola, 2015	*Discovery of the camp*, from the ‘Band of Brothers’ soundtrack and present in a scene of the television show	Liking	C		None, a historically accurate “sad narrative” of the television scene, a historically false “neutral narrative” of the television scene	90	1	This study included a piece by Debussy, without any narrative, that was used as a comparison stimulus. We do not include this as a comparison condition as there were no contextualizing conditions for this piece
Zalanowski, 1986	3 min excerpts of Berlioz's *Symphonie fantastique*, Mvt. 4; Schubert's *Symphony No. 8 in B minor*, Mvt. 2	Enjoyment	C; C	a; c	None, form free mental imagery, program note and also form imagery of described story; None, form free mental imagery, descriptive note, analytical note	60; 48	1	Due to different conditions for the two stimuli, they are categorized separately. Exp. 1 (being stimulus 1) produced significantly higher ratings for free imagery (without program note) condition only. Exp. 2 (stimulus 2) produced no significant results

a *Experiments are separated by semicolon. In some cases, there are multiple experiments in the “Result categorization” or “Understanding categorization” columns that use the same stimuli, independent variables, N, or exposure numbers. For brevity, in such cases we have not re-written these identical details*.

b *“A” denotes results strictly supporting the PHF hypothesis with significant, positive results. “A^—^” denotes significant, negative results supporting the PHF hypothesis through the “aesthetic-artistic confound” discussed in section Outline of the Psycho-historical Framework for the Science of Art Appreciation. “B” denotes inconclusive results within the same study. “C” denotes results strictly rejecting the PHF hypothesis. See section “Method and Materials” for details*.

c *Categorizations for the variable understanding are also included for studies incorporating this as a variable in relation to accompanying information. “a” denotes significantly higher ratings of understanding for conditions receiving additional contextual information. “b” denotes inconclusive results of understanding within the same study. “c” denotes no significant results indicating increased ratings of understanding for conditions receiving additional contextual information. See section “Method and Materials” for details*.

d *N refers to the overall sample population in each experiment; see main text for discussion of sample size per condition*.

e *‘Exp.’ Is the abbreviation used to refer to an experiment number within a study*.

f *To save space, Roman numeral superscript references in other columns refer to parts of this Comments column that commence with the corresponding Roman numeral superscript*.

g *For Exp. 2 of Specht (2010), both stimuli used intentionally had their accompanying artist statement switched to the opposite stimulus. We still classify this as “contextualizing information” as the participants were not aware of this switch*.

### Range of Stimuli Used in Reviewed Studies

Eleven of the 22 experiments using visual stimuli captured in this review exclusively contained abstract stimuli. Five of these 11 experiments (Jucker et al., 2014, experiments 2a and 2b; Russell, 2003, experiments 1 and 2; Russell and Milne, 1997, experiment 1) referred to the stimuli as “semi-abstract to abstract” works, and 2 experiments used surrealist works (Swami, 2013, experiments 1 and 3). Three experiments investigated representational stimuli only, whereas 8 experiments examined both abstract and representational stimuli. Of the 12 experiments using music stimuli, 7 examined classical^1^ music only, and 2 explicitly examined modern styles of music: excerpts of dance and electronic music (Anglada-Tort et al., 2018) and “immigrant music, especially the music of the Irish…[that could be] thought of as quintessentially American, such as bluegrass” (Margulis et al., 2015, p. 598). Additionally, one experiment investigated both popular and classical^1^ music (Halpern, 1992), another used an instrumental piece from the soundtrack for the television series *Band of Brothers* (Vuoskoski and Eerola, 2015), and Bradley (1972) investigated 24 “contemporary art compositions” that represented tonal, polytonal, atonal, and electronic music.

### Main Findings

Of the 34 experiments reporting results of appreciation (or equivalent) across both visual and music stimuli, 9 (26%) were categorized as “A” (supporting the PHF hypothesis), 6 (18%) were categorized as “B” (inconclusive results), and 19 (56%) were categorized as “C” (not supporting the PHF hypothesis). Two experiments categorized as A also fit the subset of A^−^ (significant negative results): one of these examined visual stimuli (Temme, 1992, experiment 4) and the second examined music stimuli (Margulis, 2010, experiment 1). For experiments exclusively examining visual stimuli, 5 out of 22 (23%) were categorized as “A,” another 4 (18%) were categorized as “B,” and 13 (59%) were categorized as “C.” For experiments exclusively examining music stimuli, 4 out of 12 experiments (33%) were categorized as “A,” 2 (17%) were categorized as “B,” and 6 (50%) were categorized as “C.” An overall analysis of these results would suggest that the majority of previous studies on this topic do not provide empirical support for the PHF hypothesis for either visual or music stimuli, however due to a number of factors (see section Discussion), this may be a highly conservative analysis and caution is advised.

Additional analysis is required on the six experiments that were categorized as “B” due to internally inconsistent results (i.e., one or more results classified as “A” and also one or more as “C” within the same experiment). Two of these studies (Halpern, 1992; Specht, 2010, experiment 1) produced results better classified in category “C” than category “A.” Halpern (1992) examined four stimuli, although only one produced a significant positive result; the remaining three stimuli produced non-significant results. Similarly, the first experiment by Specht (2010) produced a significant positive result for one of the three examined stimuli, whereas the remaining two stimuli produced non-significant results. Of the remaining four studies categorized as “B,” we first examine Damon (1933, experiment 1), which produced a significant impact on enjoyment (either positive or negative) for 45% of results, and non-significant results for the remaining 55% (see Supplementary Material). Second, Specht (2010, experiment 2) contained two stimuli (one abstract, the other representational), and produced a significant positive result (category “A”) for the representational stimulus but a non-significant result for the abstract stimulus. The remaining two experiments categorized as “B” (Swami, 2013, experiment 2; Temme, 1992, experiment 1) examined groups of abstract or representational stimuli (respectively, 8 works in each stimulus group, and 20 works in each stimulus group). Analysis was performed on groups of stimuli rather than individual works. In contrast to the results of Specht (2010, experiment 2) each experiment produced a significant positive result for their group of abstract stimuli, but did not produce a significant result for their group of representational stimuli. In summary, of the 6 experiments categorized as “B,” two could be reconceptualized as category “C” more than category “A,” whereas the remaining four experiments categorized as “B” appear to evenly represent both categories “A” and “C.”

Eleven of the 34 experiments included ratings of appreciation *as well as* ratings of understanding; 8 of these 11 experiments examined visual stimuli. For 6 of these 11 experiments, understanding ratings were categorized as “a,” meaning that in the presence of additional information the studies reported significantly higher ratings of understanding. Four of the remaining five experiments were categorized as “b,” signifying an inconclusive relationship between understanding and the presence of additional information for these studies, and 1 experiment was categorized as “c.” Examination of the four studies categorized as “b” showed that three of them (Leder et al., 2006, experiments 1 and 2; Swami, 2013, experiment 2) each evenly represented categories “a” and “c” whereas Halpern (1992) produced a result classified as “a” for one stimulus, and non-significant results (“c”) for the remaining three stimuli. As noted in section Design, these categorizations of understanding by themselves do not test the PHF hypothesis; rather they provide evidence that provision of additional information has been processed to some extent by the participant. We therefore examined the relationship between categorizations for the variables appreciation and understanding. Of these 11 experiments, 3 cases (Millis, 2001, experiment 1; Swami, 2013, experiments 1 and 3) produced significantly higher ratings of understanding for conditions receiving additional contextualization *as well as* significantly different ratings of appreciation (either positive or negative) for these contextualized conditions. These three studies support the PHF hypothesis. In addition, Swami (2013, experiment 2) reported significantly higher ratings for the contextualized condition, alongside increased ratings of understanding for this contextualized condition, for abstract stimuli only. Conversely, the representational stimuli examined here by Swami did not produce a significant effect of condition for either variable. Thus, this experiment suggests a positive and highly correlated relationship between the two variables, and we interpret the results as support for the PHF hypothesis, whereas the remaining 7 experiments did not produce such closely knit results between understanding and appreciation.

## Discussion

The initial analysis of the literature demonstrated that the majority of experiments meeting the inclusion criteria do not support the PHF hypothesis. However, due to a number of impacting factors we list here, such a conclusion lacks nuance. First, given the difficulties in applying the proposed modes of the PHF to the various approaches used in the literature, we collapsed the two later modes of the PHF into one. Second, low sample sizes and consequent low statistical power in some of the studies in the review alone may have accounted for the “C” category rather than a rejection of the PHF hypothesis. Three experiments (Cupchik et al., 1994, experiment 2; Zalanowski, 1986; Halpern, 1992, experiment 2)—one examining visual stimuli and two examining music stimuli—each contained a sample size *n* < 20 for between-subjects conditions^2^, which according to VanVoorhis and Morgan (2007) is a sample size range that could produce unacceptably low statistical power, thereby possibly masking a true underlying effect. We suggest caution in the interpretation of the results of these studies with respect to the PHF hypothesis.

Third, some caution is also required in interpretation of the reported results in section Main Findings due to the methodology used by the reviewed studies themselves. Bradley's (1972) study was categorized as “A,” however this result could have been explained in part by the confounding of the effects of additional exposures (see, e.g., Zajonc, 1968; Berlyne, 1971; Chmiel and Schubert, 2017). The study contained three conditions receiving increasing levels of contextualization (none; title and composer only; special training and experience in listening analytically), however these three conditions also received differently increased amounts of exposure alongside their increased contextualization. Specifically, those in the “none” condition received only two exposures to the stimuli, whereas those in the “title and composer” condition received five exposures, and those in the “special training” condition received 16 exposures. Finally, with the intention of interrogating the *historical understanding* of a work in Bullot and Reber's framework, the relatively small percentage (32%) of reviewed experiments containing explicit ratings of understanding is not without concern. The majority of studies in this review did not report an explicit measure of understanding, forcing us to assume that the provision of contextual information alone happened to be suitably processed by the participant. The difference in the conclusions due to “understanding measured” vs. “understanding not-measured” studies suggests additional need for caution in our conclusions, and highlights a consideration for future research to gather evidence of actual processing.

Another facet highlighted by this review is a substantial difference in the stimuli used for investigations between the two mediums. Music stimuli tended to be of a “typical” nature. Only one study (Bradley, 1972) used a style of music (atonal music—one of the four styles of music used in this study) that could be considered atypical/unusual. In contrast, 50% of the visual experiments exclusively used abstract works, with an additional 32% examining both abstract and representational works. This is noteworthy considering a hypothesis in the literature surrounding contextualization for visual works is that abstract works might be more susceptible to the effects of contextualizing information than representative works (e.g., Temme, 1992, p. 29; Leder et al., 2006, p. 179; Bordens, 2010, p. 113; Specht, 2010, p. 194; Swami, 2013, p. 286). This hypothesis reflects the increased difficulty that an individual might experience in creating their own interpretation when faced with work of a highly abstract nature. Thus, we recommend the inclusion of more atypical, unfamiliar examples of music in future research to properly investigate this possibility.

**Figure 2:**
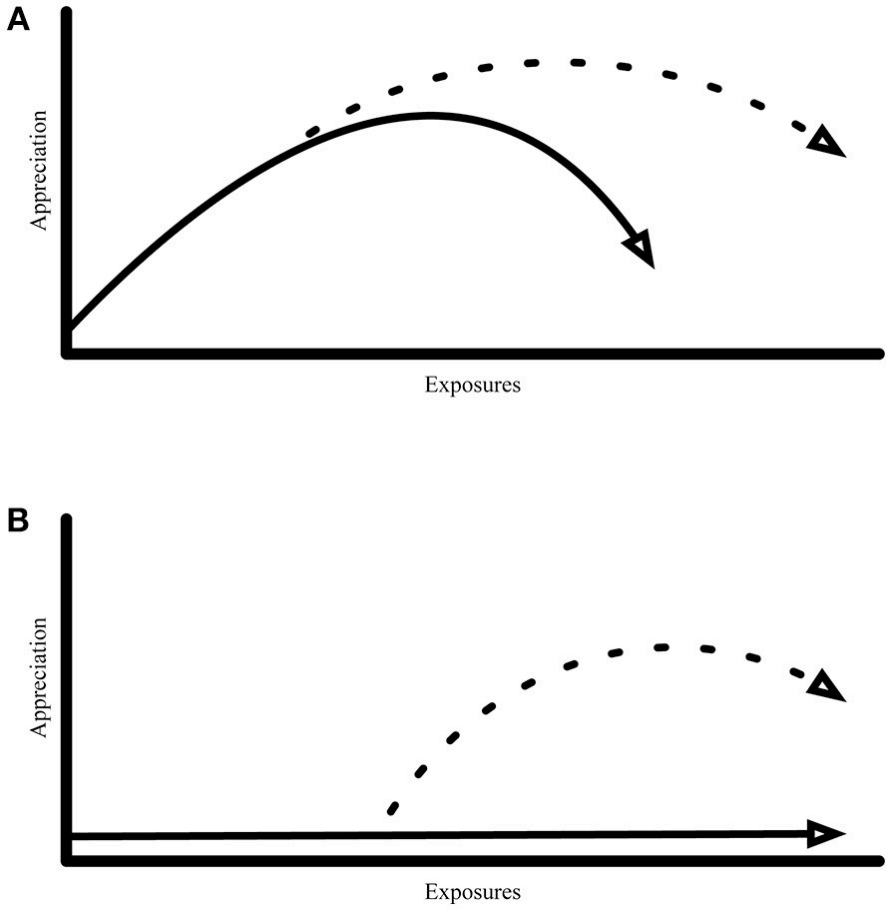
Proposed interactions between exposure and contextual information upon appreciation ratings: **(A)** The solid line represents a general appreciation trajectory that encapsulates the overarching inverted-U model due to mere exposure. The dashed line indicates the extension of this trajectory over additional exposures with the inclusion of contextualizing information about the stimulus. **(B)** The solid line represents a floor-effect trajectory, as hypothesized for examples of extreme music. The dashed line indicates the trajectory for the same stimulus as a function of exposure if contextualization is later incorporated into the experience.

A small amount of research currently exists suggesting that listeners may respond to extreme music in a notably different manner compared to “typical” styles of music. For example, while subsequent exposures to typical music stimuli will tend to produce an increase in appreciation at some point, as the first “segment” of an overall inverted-U trajectory (Heyduk, 1975; Chmiel and Schubert, 2017), in cases where the music exhibits “extreme”^3^ properties (subjectively, to the listener), this overall inverted-U trajectory appears to become less apparent (for a review, see Chmiel and Schubert, 2018a,b). While there are only a few cases in the literature examining aesthetic responses to examples of extreme music, in such cases music appreciation appears to produce a floor-effect in which it remains at or close to the minimum rating, regardless of the number of subsequent exposures (e.g., Downey and Knapp, 1927; Hargreaves, 1984). Hypothetically, this floor-effect could be a by-product of a lack of understanding of an extreme stimulus, and similarly a lack of related meaningfulness for it (see also Martindale, 1984, 1988). We tentatively suggest that appreciation for extreme examples of music might increase in the presence of contextual information to a greater degree than would be the case for music that is not extreme. For a visual outline of how the PHF hypothesis might be able to combat a floor-effect produced by an extreme example of music, see Figure 2. Alternatively, this could also be a result of appreciation for the non-extreme music already existing at a higher level, meaning that additional contextual information (if operating under the PHF hypothesis) only produces a relatively marginal impact on appreciation due to a ceiling effect (Figure 2).

## Conclusions

This paper examined the results of 23 studies (containing 34 experiments) that reported appreciation for visual or music stimuli while manipulating the amount of accompanying contextualizing information. The aim was to examine the utility of the *psycho-historical framework for the science of art appreciation* (PHF) for the two mediums, with music appreciation being a novel focus. A simplified PHF hypothesis was proposed due to small number of studies reporting both appreciation and understanding, and the difficulty in measuring complex and idiosyncratic concepts such as appreciation, understanding, and historical sensitivity through self-reporting human participants. The results were generally consistent across the two mediums, suggesting that in terms of appreciation visual and music works are responded to in a similar manner for accompanying information; when examining both mediums together 26% of studies reported significant results supporting the PHF hypothesis, yet 56% of results did not support the hypothesis, and 18% produced inconclusive results within the same experiment. In addition to the above analysis, we examined the subset of eleven studies that reported ratings of both appreciation *and* understanding. This allowed a more nuanced consideration of the PHF, by allowing us to test whether increased understanding brings with it changed appreciation. Thirty-six percent of these experiments produced results supporting the PHF hypothesis (such as reporting significant change in appreciation alongside increases in understanding for conditions receiving additional contextualization). Therefore, the majority of results in the literature do not support the simplified PHF hypothesis for either medium. However, other factors may be at play that indicate why the PHF has yet to receive sufficient empirical consideration.

The continuing investigation on why we prefer certain works of art, music, and the like has fascinated psychologists since the beginnings of experimental psychology (see, e.g., Fechner, 1876/1997), yet the consensus in the field remains divided. One possible cause of this may lie in the general movement of aesthetics toward ecologically-based explanations since the 1980s, as highlighted in Figure 1 (but see, also Hargreaves and North, 2010). This is a noteworthy shift considering that any impact of ecological variables may prove difficult to determine in parsimonious terms due to their subjective, idiosyncratic nature. In contrast, recent evidence (Chmiel and Schubert, 2017, 2018b) suggests that for music appreciation (preference in particular), collative variables such as exposure are able to predict general listening tendencies through an overarching inverted-U trajectory with substantial efficacy. If a sizeable portion of appreciation can be explained by collative variables, which could be thought of as the building blocks of appreciation, this may explain why contextualization does not always produce a significant impact; the PHF may be active, but there is less variance left to explain. Therefore, controlled manipulation of collative and ecological variables together may prove a fruitful avenue for developing a more robust understanding of appreciation (e.g., Hargreaves and North, 2010; Hargreaves, 2012; Schubert et al., 2014). However, in reality collative variables have been largely overlooked in recent decades (Martindale et al., 1990; North and Hargreaves, 2000; Silvia, 2005); (Chmiel and Schubert, 2017).

The potential utility of such a joint approach cannot be overstated. Where collative variables may provide a general foundation for appreciation tendencies, as noted above, they may also be limited in specific circumstances—specifically those entailing individual, personal experiences. Based on a liberal interpretation of the literature investigated, collative variables and context interactively influence stimulus appreciation. Evidence exists showing that strong positive association may outweigh some of the negative effects of over-exposure (Davies, 1978; Martindale, 1988; Chartrand and Dalton, 2009; Schubert et al., 2014). Therefore, as suggested in Figure 2, the inverted-U effect due to exposure may interact with contextual information by extending the unfolding of the effect, while retaining a strong overlap in the rising portion of the curve (see Figure 2A), explaining why at times contextual conditions made little difference to already liked music and art. But in cases where instead of the inverted-U, a floor effect of appreciation remains regardless of exposure to a disliked, extreme stimulus, the presence of contextual influence may ignite the more regular inverted-U trajectory, an interaction that is borne out by the small number of studies that have examined extreme stimuli and the value to appreciation that the addition of context provides (Figure 2B).

To summarize, if support for the PHF hypothesis was hidden from our analysis of the literature, four factors explain why:

1. Methodological limitations such as non-significant results reported in studies with small participant sizes;
2. Variance in appreciation response is already explained by variables such as exposure, meaning that the PHF hypothesis may be active but only able to make a small contribution;
3. In relation to factor two—the effect of hidden interactions—the PHF hypothesis may be easier to demonstrate when the stimuli under contemplation are extreme/unusual/unfamiliar;
4. Difficulty in translating philosophically-based theory into empirical practice, for example quantifying complex concepts such as appreciation and understanding from self-reporting human participants.

Bullot and Reber's framework therefore serves an important role in further nuancing our understanding of art appreciation beyond the lens of empirical psychology research. Such an approach is largely untapped because many approaches steeped in the methodologies of psychology and neuroscience tend to overlook the influence of historical elements rather than integrating them. Thus, the present study highlights a number of difficulties in quantifying the efficacy of the historical approach and suggests ways forward for testing the full potential of the framework in explaining art appreciation.

## Author Contributions

AC designed, collected, analyzed the data. AC drafted and refined the manuscript. ES had overall oversight of the project, and worked with AC in refining issues concerned with design, analysis, and manuscript writing.

### Conflict of Interest Statement

The authors declare that the research was conducted in the absence of any commercial or financial relationships that could be construed as a potential conflict of interest.

## Footnotes

^1^Referring to classical music of the common practice period.

^2^Here we do not include studies containing within-subject designs with lower sample-size (e.g., Margulis, 2010) as such designs boost statistical power in comparison to between-subject designs (Charness et al., 2012; Bellemare et al., 2014).

^3^We refer to the “abstractness” of the stimuli as “unusualness,” being a proposed collative variable (see Chmiel and Schubert, 2018a,b).

## References

[B1] Anglada-Tort M. Müllensiefen D. (2017). The repeated recording illusion: the effects of extrinsic and individual difference factors on musical judgement. Music Percept. 35, 94–117. 10.1525/mp.2017.35.1.94

[B2] Anglada-Tort M. Steffens J. Müllensiefen D. (2018). Names and titles matter: the impact of linguistic fluency and the affect heuristic on aesthetic and value judgements of music. Psychol. Aesthetics Creativ. Arts. 10.1037/aca0000172

[B3] Belke B. Leder H. snd Augustin M. D. (2006). Mastering style-effects of explicit style-related information, art knowledge and affective state on appreciation of abstract paintings. Psychol. Sci. 48, 115–134.

[B4] Bellemare C. Bissonnette L. Kröger S. (2014). Statistical power of within and between-subjects designs in economic experiments. Cahier Recherche Working Paper 14, 1–25. 10.2139/ssrn.3149007

[B5] Berlyne D. E. (1971). Aesthetics and Psychobiology. New York, NY: Appleton-Century-Crofts.

[B6] Bordens K. S. (2010). Contextual information, artistic style and the perception of art. Empir. Stud. Arts 28, 111–130. 10.2190/EM.28.1.g

[B7] Bradley I. L. (1972). Effect on student musical preference of a listening program in contemporary art music. J. Res. Music Educ. 20, 344–353. 10.2307/3343887

[B8] Bullot N. J. Reber R. (2013a). The artful mind meets art history: toward a psycho-historical framework for the science of art appreciation. Behav Brain Sci. 36, 123–180. 10.1017/S0140525X12000489 23507091

[B9] Bullot N. J. Reber R. (2013b). A psycho-historical research program for the integrative science of art. Behav. Brain Sci. 36, 163–180. 10.1017/S0140525X12002464 23617023

[B10] Bullot N. J. Reber R. (2017). Artistic misunderstandings: the emotional significance of historical learning in the arts. Behav. Brain Sci. 40:E354. 10.1017/S0140525X17001625 29342775

[B11] Charness G. Gneezy U. Kuhn M. A. (2012). Experimental methods: between-subject and within-subject design. J. Econ. Behav. Organ. 81, 1–8. 10.1016/j.jebo.2011.08.009

[B12] Chartrand T. L. Dalton A. N. (2009). Mimicry: its ubiquity, importance, and functionality, in Oxford Handbook of Human Action, eds Morsella E. Bargh J. A. Gollwitzer P. M (New York, NY: Oxford University Press), 458–483.

[B13] Chmiel A. Schubert E. (2017). Back to the inverted-U for music preference: a review of the literature. Psychol. Music 45, 886–909. 10.1177/0305735617697507

[B14] Chmiel A. Schubert E. (2018a). Emptying rooms: when the inverted-U model of preference fails—an investigation using music with collative extremes. Empir. Stud. Arts 36, 199–221. 10.1177/0276237417732683

[B15] Chmiel A. Schubert E. (2018b). Unusualness as a predictor of music preference. Musicæ Sci. 1029864917752545. 10.1177/1029864917752545

[B16] Cleeremans A. Ginsburgh V. Klein O. Noury A. (2016). What's in a name? the effect of an artist's name on aesthetic judgments. Empir. Stud. Arts 34, 126–139. 10.1177/0276237415621197

[B17] Cupchik G. C. Shereck L. Spiegel S. (1994). The effects of textual information on artistic communication. Vis. Arts Res. 20, 62–78.

[B18] Damon K. F. (1933). Program Notes for the Listener to Music: A Study of Their Development and Effect Upon the Listener's Reactions to Unfamiliar Music. New York, NY: Freybourg Printing Co.

[B19] Davies J. B. (1978). The Psychology of Music. London: Hutchinson.

[B20] Downey J. E. Knapp G. E. (1927). The effect on a musical programme of familiarity and of sequence of selections, in The Effects of Music: A Series of Essays, ed Schoen M (New York, NY: Books For Libraries Press), 223–243.

[B21] Duerksen G. L. (1972). Some effects of expectation on evaluation of recorded musical performance. J. Res. Music Educ. 20, 268–272. 10.2307/3344093

[B22] Fechner G. T (1876/1997). Various attempts to establish a basic form of beauty: experimental aesthetics, golden section and square. Empir. Stud. Arts 15, 115–129. 10.2190/DJYK-98B8-63KR-KUDN

[B23] Halpern J. (1992). Effects of historical and analytical teaching approaches on music appreciation. J. Res. Music Educ. 40, 39–46. 10.2307/3345773

[B24] Hargreaves D. J. (1984). The effects of repetition on liking for music. J. Res. Music Educ. 32, 35–47. 10.2307/3345279

[B25] Hargreaves D. J. (2012). Musical imagination: perception and production, beauty and creativity. Psychol. Music 40, 539–557. 10.1177/0305735612444893

[B26] Hargreaves D. J. North A. C. (2010). Experimental aesthetics and liking for music, in Handbook of Music and Emotion: Theory, Research, Applications, eds Juslin P. N. Sloboda J (New York, NY: Oxford University Press), 515–546.

[B27] Herzog M. Lepa S. Egermann H. Steffens J. Schönrock A. (2017). Predicting musical meaning in audio branding scenarios, in Paper presented at the Proceedings of the 25th Anniversary Conference of the European Society for the Cognitive Sciences of Music (Ghent: ESCOM).

[B28] Heyduk R. G. (1975). Rated preference for musical compositions as it relates to complexity and exposure frequency. Percept. Psychophys. 17, 84–91. 10.3758/BF03204003

[B29] Jucker J. L. Barrett J. L. Wlodarski R. (2014). “I just don't get it”: perceived artists' intentions affect art evaluations. Empir. Stud. Arts 32, 149–182. 10.2190/EM.32.2.c

[B30] Kirk U. Skov M. Hulme O. Christensen M. S. Zeki S. (2009). Modulation of aesthtic value by semantic context: an fMRI study. Neuroimage 44, 1125–1132. 10.1016/j.neuroimage.2008.10.009 19010423

[B31] Kroger C. Margulis E. H. (2016). “But they told me it was professional”: extrinsic factors in the evaluation of musical performance. Psychol. Music 45, 49–64. 10.1177/0305735616642543

[B32] Kruger J. Wirtz D. Van Boven L. Altermatt T. W. (2004). The effort heuristic. J. Exp. Soc. Psychol. 40, 91–98. 10.1016/S0022-1031(03)00065-9

[B33] Leder H. Carbon C. C. Ripsas A. L. (2006). Entitling art: influence of title information on understanding and appreciation of paintings. Acta Psychol. 121, 176–198. 10.1016/j.actpsy.2005.08.005 16289075

[B34] Margulis E. H. (2010). When program notes don't help: music descriptions and enjoyment. Psychol. Music 38, 285–302. 10.1177/0305735609351921

[B35] Margulis E. H. Kisida B. Greene J. P. (2015). A knowing ear: the effect of explicit information on children's experience of a musical performance. Psychol. Music 43, 596–605. 10.1177/0305735613510343

[B36] Martindale C. (1984). The pleasures of thought: a theory of cognitive hedonics. J. Mind Behav. 5, 49–80.

[B37] Martindale C. (1988). Aesthetics, psychobiology, and cognition, in The Foundations of Aesthetics, Art, and Art Education, eds Farley F. H. Neperud R. W (New York, NY: Praeger Publishers), 7–42.

[B38] Martindale C. Moore K. Borkum J. (1990). Aesthetic preference: anomalous findings for Berlyne's psychobiological theory. Am. J. Psychol. 103, 53–80. 10.2307/1423259

[B39] Millis K. (2001). Making meaning brings pleasure: the influence of titles on aesthetic experiences. Emotion 1, 320–329. 10.1037/1528-3542.1.3.320 12934689

[B40] North A. C. Hargreaves D. J. (2000). Collative variables versus prototypicality. Empir. Stud. Arts 18, 13–17. 10.2190/K96D-085M-T07Y-61AB

[B41] Prince W. F. (1974). Effects of guided listening on musical enjoyment of junior high school students. J. Res. Music Educ. 22, 45–51. 10.2307/3344617

[B42] Rigg M. G. (1948). Favorable versus unfavorable propaganda in the enjoyment of music. J. Exp. Psychol. 38, 78–81. 10.1037/h0056077 18910260

[B43] Russell P. A. (2003). Effort after meaning and the hedonic value of paintings. Br. J. Psychol. 94, 99–110. 10.1348/000712603762842138 12648392

[B44] Russell P. A. Milne S. (1997). Meaningfulness and hedonic value of paintings: effects of titles. Empir. Stud. Arts 15, 61–73. 10.2190/EHT3-HWVM-52CB-8QHJ

[B45] Schubert E. Hargreaves D. J. North A. C. (2014). A dynamically minimalist cognitive explanation of musical preference: is familiarity everything? Front. Psychol. 5:38. 10.3389/fpsyg.2014.00038 24567723 PMC3915416

[B46] Silvia P. J. (2005). Emotional responses to art: from collation and arousal to cognition and emotion. Rev. Gen. Psychol. 9, 342–357. 10.1037/1089-2680.9.4.342

[B47] Smith L. F. Bousquet S. G. Chang G. Smith J. K. (2006). Effects of time and information on perception of art. Empir. Stud. Arts 24, 229–242. 10.2190/DJM0-QBDW-03V7-BLRM

[B48] Specht S. M. (2010). Artist's statements can influence perceptions of artwork. Empir. Stud. Arts 28, 193–206. 10.2190/EM.28.2.e

[B49] Steinbeis N. Koelsch S. (2009). Understanding the intentions behind man-made products elicits neural activity in areas dedicated to mental state attribution. Cereb. Cortex 19, 619–623. 10.1093/cercor/bhn110 18603608

[B50] Swami V. (2013). Context matters: investigating the impact of contextual information on aesthetic appreciation of paintings by Max Ernst and Pablo Picasso. Psychol. Aesthetics Creativ. Arts 7, 285–295. 10.1037/a0030965

[B51] Temme J. E. V. (1992). Amount and kind of information in Museums: its effects on visitors satisfaction and appreciation of art. Vis. Arts Res. 18, 28–36.

[B52] VanVoorhis C. W. Morgan B. L. (2007). Understanding power and rules of thumb for determining sample sizes. Tutorials Quant. Methods Psychol. 3, 43–50. 10.20982/tqmp.03.2.p043

[B53] Vuoskoski J. K. Eerola T. (2015). Extramusical information contributes to emotions induced by music. Psychol. Music 43, 262–274. 10.1177/0305735613502373

[B54] Zajonc R. B. (1968). Attitudinal effects of mere exposure. J. Personal. Soc. Psychol. Monogr. 9, 1–27. 10.1037/h0025848

[B55] Zalanowski A. H. (1986). The effects of listening instructions and cognitive style on music appreciation. J. Res. Music Educ. 34, 43–53. 10.2307/3344797

