# Supplementary material for: Psycho-Historical Contextualization for Music and Visual Works: A Literature Review and Comparison Between Artistic Mediums
Source: Front Psychol. 2019 Feb 19;10:182. doi: 10.3389/fpsyg.2019.00182 (PMC6389697; doi:10.3389/fpsyg.2019.00182)
Supplement: Supplementary file 1 [file Data_Sheet_1.pdf]

Supplementary Material for

Psycho-historical contextualization for music and visual works: a literature review and comparison between artistic mediums

doi: 10.3389/fpsyg.2019.00182

Anthony Chmiel, Emery Schubert

Empirical Musicology Laboratory, School of the Arts and Media, University of New South Wales, Sydney, NSW, Australia

Correspondence:
Emery Schubert
e.schubert@unsw.edu.au

Before the studies in this review were categorized, we performed additional analyses on three experiments: Damon (1933, experiments 1 and 2), which contained music stimuli, and Swami (2013, experiment 2), which contained visual stimuli. The reasons for re-analyses are discussed below. In the first of these re-analyzed studies, Damon (1933) examined orchestral, solo violin, and vocal ensemble music. In what we refer to as experiment 1 (pp. 32-60), 120 participants were exposed to 12 pieces and received either a) a short program note containing relevant information on the music and/or performance, such as technical or compositional elements, and also containing a suggestion for story-like or emotional meaning, b) the title and composer of the stimulus only, or c) no information at all. We henceforth refer to these three conditions as *with*, *title*, and *without*. Enjoyment responses were recorded on 4-point scales for each piece, and Damon reported that for 8 of the 12 pieces participants gave higher enjoyment ratings when receiving the *with* condition than the *without* condition. Damon only directly compared the *with* and *without* conditions; he investigated the *title* condition separately in a following section, albeit in response to the same stimuli as the previous two conditions. The raw data were reported (pp. 52-60) although no inferential statistical analyses were performed. We conducted inferential analysis on all three conditions. The reported means for each stimulus, separated by condition, are plotted in Supplementary Figure 1. We omitted the piece *Fireworks* by Stravinsky as a number of enjoyment ratings for the *with* condition were missing from the reported data.

We conducted a mixed-design ANOVA on Damon's reported enjoyment ratings (dependent variable) with *piece* as a within-subjects factor, and *condition* (with, without, or title) as a between-subjects factor. Greenhouse-Geisser correction was used when departures from sphericity were observed. There were significant main effects for *piece* ($F(5,1674) = 95.95$, $p < .001$, $\eta^2 = .212$) and *condition* ($F(2,357) = 14.45$, $p < .001$, $\eta^2 = .075$), and a significant interaction for *piece* by *condition* ($F(9,1674) = 10.21$, $p < .001$, $\eta^2 = .054$). Post hoc independent-samples *t*-test results are reported in

Supplementary Table 1, with Holm-Bonferroni sequential correction. When comparing the conditions *with* and *without*, 4 out of 11 stimuli (36%) produced significant differences; the *with* condition produced a higher enjoyment rating for 3 of these significant comparisons (27% of the total results). When comparing the conditions *title* and *without*, 4 out of 11 stimuli (36%) produced significant results; the *title* condition produced a higher enjoyment rating than the *without* condition for only 1 of these 4 significant results (9%). Finally, when comparing the conditions *with* and *title*, 7 out of 11 stimuli (64%) produced significant results; the *with* condition produced a higher enjoyment rating than the *title* condition for 6 of these 7 significant results (54% of the total results). In total, 15 of 33 cases (45%) produced significant results, with 10 of these (30% of all cases) outlining positive effects of contextualization on enjoyment. As the PHF encapsulates both positive and negative impact of contextualization on preference (see main article, section *Outline of the Psycho-historical framework for the science of art appreciation*), all of these significant results support the PHF hypothesis, whereas the remaining 55% of results do not. We therefore denote this first experiment as Category 'B' (inconclusive results) in Table 1 of the main article.

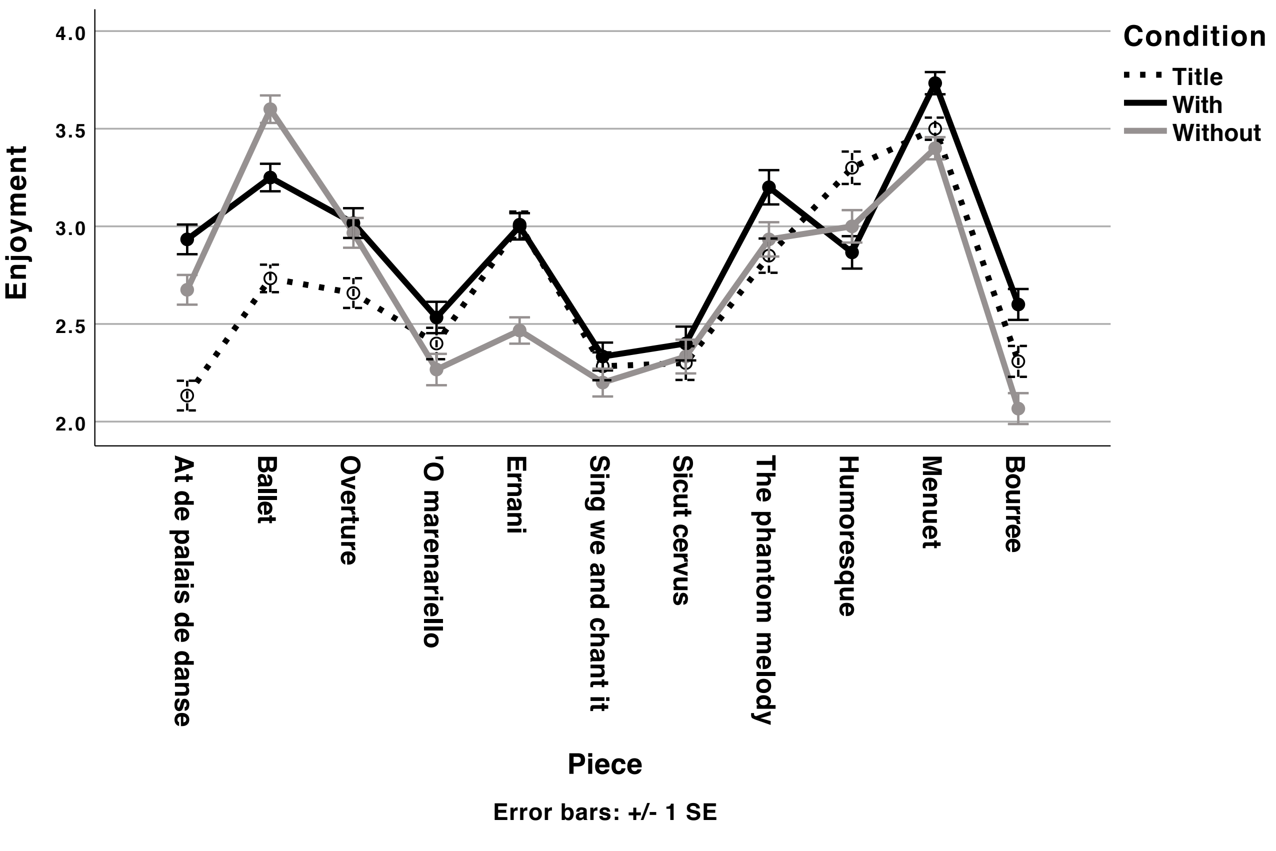

Supplementary Figure 1. Mean and standard error of enjoyment ratings by listening condition, based on raw data reported by Damon (1933, experiment 1). The piece *Fireworks* was omitted as a number of responses were missing for the *with* condition.

Supplementary Table 1. *Post hoc independent samples t-test results for enjoyment between listening conditions, created from descriptive statistics reported in Damon (1933, experiment 1). Tests have been subjected to Holm-Bonferroni sequential correction. Cohen's d is reported only for results that reached significance (p < .05) before correction*

Piece	With vs. Without		Title vs. Without		With vs. Title	
	p	*d*	*p*	*d*	*p*	*d*
At de palais de danse	.234	.25	<.001 -	.71	<.001 +	1.14
Ballet	<.001 -	.56	<.001 -	1.05	<.001 +	.62
Overture	>.999		.04 -	.37	<.001 +	.48
'O marenariello	.133	.30	>.999		.247	
Ernani	<.001 +	.69	<.001 +	.69	>.999	
Sing we and chant it	.728		>.999		>.999	
Sicut cervus	>.999		>.999		>.999	
The phantom melody	.234	.27	>.999		.03 +	.36
Humoresque	.858		.084	.33	<.001 -	.51
Menuet	<.001 +	.55	>.999	.19	.014 +	.40
Bourree	<.001 +	.58	.276	.26	.018 +	.39

Note. The piece *Fireworks* was omitted, as a number of responses were missing for the *with* condition. + denotes a significant positive impact on enjoyment, whereas – denotes a significant negative impact.

In what we refer to as experiment 2 (pp. 61-67), Damon investigated the effects of the type of information given to participants. Using a new sample of 130 participants, Damon exposed them to 3 of the previous 12 stimuli from experiment 1 (*Fireworks*, *Ernani*, and *The phantom melody*), and participants again rated enjoyment on a 4-point scale. All participants were given program notes, however half of the program notes focused solely on intrinsic elements from the composition and/or performance, whereas the other half focused solely on story-like and emotional elements. Therefore, this experiment did not meet the inclusion requirements for the review and is not categorized in Table 1 of the main article (*Results* section). However, as Damon again only reported descriptive statistics and raw data (p. 63), we decided to plot the reported means, separated by listening condition (see Supplementary Figure 2), and to examine the results through a mixed-design ANOVA, as above. The two conditions will henceforth be referred to as *descriptive* and *story*. There were significant main effects for *piece* ($F(2,488) = 183.95$, $p < .001$, $\eta^2 = .416$) and *condition* ($F(1,258) = 19.41$, $p < .001$, $\eta^2 = .070$); there was also a significant interaction for *piece* by *condition* ($F(2,488) = 3.97$, $p = .022$, $\eta^2 = .015$).

As with experiment 1, all post hoc tests were subjected to Holm-Bonferroni sequential correction. For *Fireworks*, the *descriptive* condition reported a mean enjoyment rating of 1.98 (*SD* = 0.71), compared to a mean rating of 1.30 (*SD* = 0.57) for the *story* condition. A post hoc independent samples *t*-test produced no significant difference in enjoyment ratings for this stimulus ($t(258)$ = 0.58, p = .564, d = .078). For *Ernani*, the *descriptive* condition reported a mean enjoyment rating of 2.04 (*SD* = 0.75), compared to a mean rating of 2.41 (*SD* = 1.05) for the *story* condition. This difference in enjoyment was significant ($t(258)$ = 3.25, p = .003, d = .404). For *The phantom melody*, the *descriptive* condition had a mean enjoyment rating of 3.20 (*SD* = 0.88), compared to a mean rating of 3.44 (*SD* = 0.61) for the *story* condition. This difference in enjoyment was significant ($t(258)$ = -2.53, p = .024, d = .316). Therefore, for two of the three stimuli, the story-like condition produced significantly higher ratings of enjoyment than the technical description.

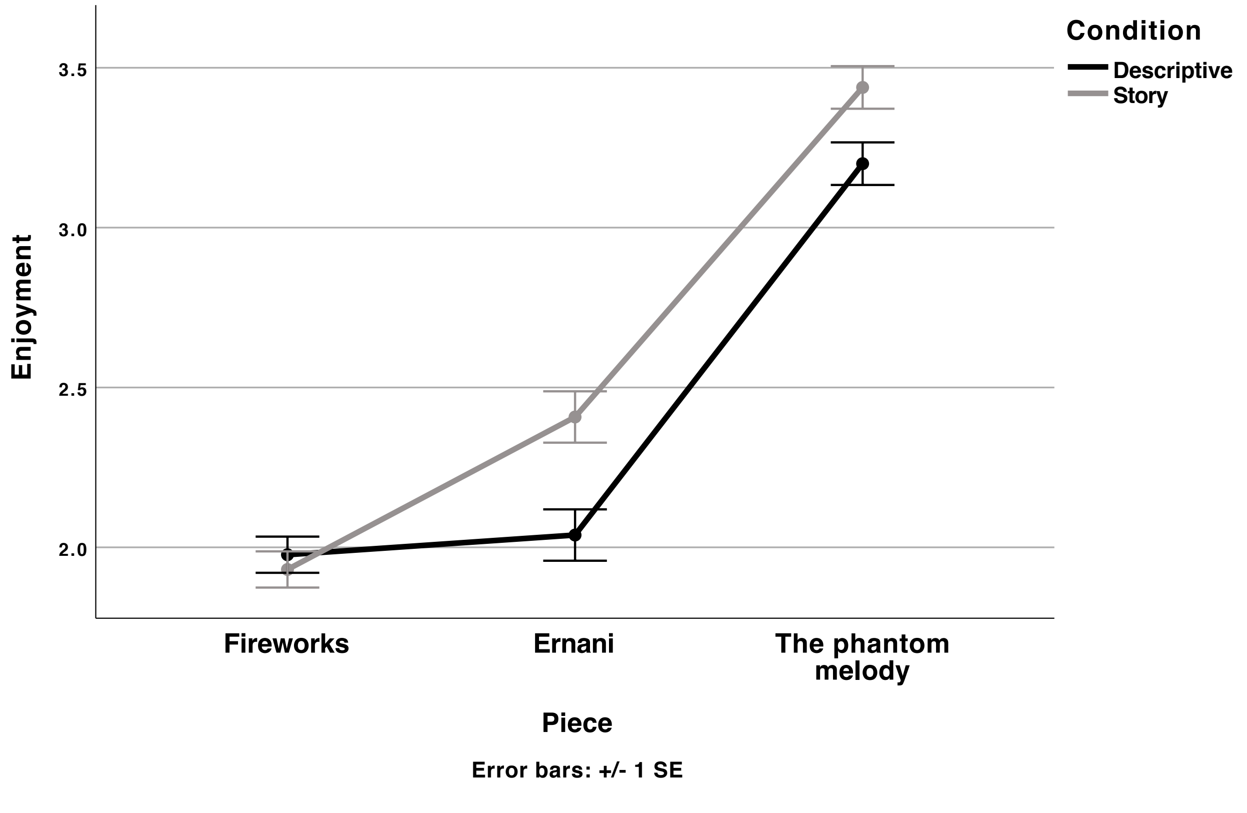

Supplementary Figure 2. Mean enjoyment ratings separated by listening condition, based on raw data reported by Damon (1933, experiment 2).

In the final re-analyzed study (Swami, 2013, experiment 2), participants rated aesthetic appreciation for either abstract paintings from Picasso's 'analyst cubist period', or representational paintings from Picasso's 'blue period'. Additionally, participants received either content-specific contextualization (the time span of the designated period, Picasso's typical approaches to works in this period, and some suggested elements to look for), or no information at all (henceforth referred to as *control*). For the abstract stimuli Swami reported significantly higher appreciation ratings for the contextualized condition compared to the *control*, although no significant effects of condition are reported for the representational stimuli. Interestingly, the accompanying *t*-test (p. 291) for the representational stimuli (described as producing no significant difference in condition) contains a significant p value ($p < .001$, $d = .33$). In order to check this conflicting report, we decided to perform an independent-samples *t*-test on the reported *M*, *SD*, and *N* data for these two conditions of the representational stimuli. Swami's initial analysis (p. 290) reports a Bonferroni corrected significance threshold of $p < .008$; therefore the results of our analysis did not reach significance in terms of this correction value ($t(69) = -2.68$, $p = .009$, $d = .636$), although we also note the relatively large effect size. Regardless, we categorize this study overall as 'B' (inconclusive results) in Table 1 of the main article, which corresponds with Swami's interpretation.
